# Comparative performance of contemporary multimodal large language models in retinal imaging question answering

**DOI:** 10.3389/fmed.2026.1934968

**Published:** 2026-08-20

**Authors:** Xiaochen Gu, Yizhou Yang, Xuanqiao Lin, Xu Chen

**Affiliations:** 1 National Clinical Research Center for Ocular Diseases, Eye Hospital, Wenzhou Medical University, Wenzhou, China; 2 Department of Ophthalmology, Eye, Ear, Nose, and Throat Hospital of Fudan University, Shanghai, China

**Keywords:** artificial intelligence, diagnostic accuracy, large language models, ophthalmology, optical coherence tomography, retinal disease

## Abstract

**Background:**

Contemporary multimodal large language models (LLMs) can process both clinical text and medical images, but their reliability in retinal imaging-based question answering remains uncertain. This study evaluated the performance of six contemporary multimodal LLMs on retinal multiple-choice questions derived from OCTCases.

**Methods:**

This cross-sectional benchmark study included 226 multiple-choice questions from 78 OCTCases retinal cases, comprising 151 image-based and 75 nonimage-based questions. Six multimodal LLMs were evaluated: ChatGPT-5.5 Instant, ChatGPT-5.5 Thinking, Grok 4, Gemini 3, DeepSeek V4, and Kimi 2.5. Model-selected answers were compared with the OCTCases answer key, which was reviewed by three retina specialists. Accuracy was assessed overall and by question subset. Pairwise model comparisons, inter-model agreement, voting-based group-answer performance, item difficulty distribution, and interface latency were analyzed.

**Results:**

Overall accuracy ranged from 59.3 to 77.4%, with the highest accuracy observed for ChatGPT-5.5 Thinking, followed by Gemini 3 and ChatGPT-5.5 Instant. DeepSeek V4 showed the lowest overall accuracy. All models performed better on nonimage-based questions than on image-based questions. In the image-based subset, Gemini 3 achieved the highest accuracy, whereas DeepSeek V4 showed the lowest accuracy. Under the majority-vote rule, group answers were generated for 182 of 226 questions and achieved an accuracy of 89.6% among questions with a determinate group answer. The plurality-vote rule generated group answers for more questions but with lower accuracy. Inter-model agreement was generally higher for nonimage-based than image-based questions, and all questions answered incorrectly by all six models were image-based. Interface latency varied across models and showed no consistent association with response correctness.

**Conclusion:**

Contemporary multimodal LLMs showed promising but uneven performance on OCTCases retinal questions. Their performance was consistently better on nonimage-based than image-based questions, indicating that retinal image interpretation remains a major limitation. Voting-based group answers may provide a useful reliability signal when model agreement is present, whereas model disagreement may help identify difficult or visually ambiguous questions requiring specialist review. These findings support continued benchmarking and cautious, specialist-supervised use of multimodal LLMs in retinal education and structured imaging question answering.

## Introduction

Artificial intelligence (AI) has become increasingly prominent in medicine, particularly in areas that require interpretation of complex clinical information and medical images (1). Ophthalmology is one of the most image-dependent medical specialties, and retinal disease assessment relies heavily on optical coherence tomography (OCT), fundus photography, fluorescein angiography, and optical coherence tomography angiography (OCTA) (2). Among these modalities, optical coherence tomography is especially important because it provides high-resolution cross-sectional information about retinal morphology, enabling clinicians to identify subtle structural abnormalities, monitor disease activity, and guide management decisions (3, 4).

The rapid development of large language models (LLMs) has further expanded the potential role of artificial intelligence in ophthalmology (5, 6). Early LLMs were primarily designed for text-based interaction, but newer multimodal models can process both written clinical information and images (7). This evolution is particularly relevant to retinal disease assessment, where accurate interpretation often requires combining patient history, visual symptoms, multimodal imaging features, and disease-specific knowledge (8). However, whether current multimodal LLMs can consistently handle image-centered ophthalmic questions remains uncertain.

Previous studies have suggested that LLMs may perform well on ophthalmic knowledge assessments and structured clinical questions (9, 10). Nevertheless, visual interpretation appears to remain more challenging than text-based reasoning. In retinal imaging, small morphological differences on OCT may distinguish entities such as intraretinal fluid, subretinal fluid, epiretinal membrane, macular hole, retinoschisis, or choroidal neovascularization. Errors in recognizing these features may directly affect the selected diagnosis, recommended investigation, or management option (11, 12). Therefore, retinal OCT–based question answering provides a useful setting to examine the strengths and limitations of contemporary multimodal LLMs.

Most existing evaluations of LLMs in ophthalmology have focused mainly on aggregate accuracy (13, 14). Although accuracy is important, it does not fully describe model behavior, particularly when models are tested on tasks that differ in their reliance on visual interpretation. Moreover, comparisons based only on individual model performance may overlook cross-model agreement and the potential value of multi-model consensus when different models show complementary answer patterns (15). Therefore, in this study, we evaluated six contemporary multimodal LLMs using multiple-choice retinal questions derived from OCTCases, assessing model-level accuracy together with modality-specific performance, paired model differences, inter-model agreement, consensus behavior, and exploratory interface latency patterns in retinal optical coherence tomography-related question answering.

## Methods

### Study design and data source

This cross-sectional benchmark study evaluated the performance of contemporary multimodal LLMs on retinal multiple-choice questions derived from the OCTCases educational platform^1^. OCTCases provides structured ophthalmic cases that include clinical stems, retinal imaging, multiple-choice questions, and platform-provided answer keys. All available multiple-choice questions from the retina section of OCTCases were screened for inclusion. A total of 226 questions from 78 retinal cases were included, and no eligible question was excluded.

The OCTCases answer key was used as the primary reference standard. The answer key and question classifications were reviewed by three retina specialists, each with at least 5 years of clinical experience in retinal disease and multimodal retinal imaging interpretation. The reviewers were blinded to model responses during the review process. Disagreements regarding the reference answer, image-based classification, question type, or disease/topic category were resolved through discussion until consensus was reached. Formal inter-reviewer agreement was not calculated.

Each question was classified as image-based or nonimage-based according to whether retinal imaging was required to determine the correct answer. Image-based questions required interpretation of OCT, fundus photography, fluorescein angiography, OCTA, or multimodal retinal imaging. Nonimage-based questions could be answered using the clinical stem, question, and answer options alone. When both clinical and imaging information were present, the question was classified as image-based if direct interpretation of the imaging findings was required to distinguish the correct answer from the distractors. Of the 226 questions, 151 were image-based and 75 were nonimage-based.

For image-based questions, all images associated with the corresponding question were provided to the models whenever available. When a question contained multiple related images, the relevant images were uploaded together with the clinical stem, question, and answer options. Images were uploaded without intentional cropping, annotation, compression, contrast adjustment, or rearrangement. For nonimage-based questions, only the clinical stem, question, and answer options were provided.

The included questions were further categorized according to question type and disease/topic category. Question type was assigned according to the main cognitive task required to answer each question, such as imaging finding or anatomy recognition, diagnosis, treatment or management, investigation, staging or etiology, or other knowledge-based reasoning. Disease/topic categories were assigned according to the primary retinal condition or dominant clinical theme tested by each question. The distribution of question types and disease/topic categories is summarized in Supplementary Table S2.

### Model selection and testing configuration

Six contemporary multimodal LLMs were evaluated: ChatGPT-5.5 Instant, ChatGPT-5.5 Thinking, Grok 4, Gemini 3, DeepSeek V4, and Kimi 2.5. All models were accessed through their publicly accessible user-facing web interfaces rather than application programming interfaces (APIs). Testing was conducted between 8 June 2026 and 14 June 2026 using the same computer, browser, and internet access environment. The model names reported in this study correspond to the names displayed in the respective web interfaces during the testing period. Account or subscription levels, reasoning or thinking mode settings, and access dates are summarized in Supplementary Table S1. Except for ChatGPT-5.5 Instant, all models were evaluated using their available reasoning or thinking mode. Because temperature and other sampling parameters were not manually adjustable in these public web interfaces, no custom sampling settings were applied.

Each model answered each question three times. For each run, the question was tested in a new chat session or after clearing the previous conversation history, and the first complete response in that run was recorded for analysis. No prior conversation context was retained between runs or between questions. Custom instructions, memory functions, web browsing, code execution, retrieval tools, external tools, follow-up prompts, and iterative refinements were not used.

The prompt followed the original OCTCases structure and consisted of the case identifier, patient presentation, relevant clinical information, question text, and answer options, without additional explanation, coaching, or task-specific prompt engineering. For image-based questions, the associated retinal images were uploaded together with the corresponding case text and question. Images were uploaded as PNG files at their original saved resolutions; image resolution was not manually modified or recorded. When several images were available for a question, they were uploaded in the same order as displayed on OCTCases. No image was intentionally cropped, annotated, compressed, contrast-adjusted, enhanced, or rearranged before upload. The questions were tested in the original OCTCases order, and the order of model testing was not randomized. The exact prompt structure and image-upload procedure are provided in Supplementary Methods.

### Outcome assessment

For each run, the final answer selected by each model was extracted from its response and compared with the OCTCases answer key. When a model clearly selected a single option, that option was recorded as the run-level answer. Responses were classified as incorrect if the model selected more than one option, provided an ambiguous answer, refused to answer, or failed to identify a single final option. Each model answered each question three times in independent runs. For each model–question pair, the final model-level answer was determined by majority vote across the three runs. When at least two of three runs selected the same option, that option was used as the final model-level answer. If the three runs selected three different options or no determinate majority answer was available, the model-level answer was classified as incorrect for the primary accuracy analysis.

The primary outcome was answer accuracy, defined as the proportion of questions for which the final model-level answer matched the OCTCases answer key. Accuracy was assessed for the full question set and separately for image-based and nonimage-based subsets. All primary accuracy, pairwise comparison, inter-model agreement, voting-based group-answer, and item-difficulty analyses were based on the final model-level answers derived from the three-run majority rule. To evaluate whether agreement across models could improve answer reliability, two predefined voting rules were applied across the six models. The majority-vote rule required at least four of six models to select the same answer. The plurality-vote rule required one answer to receive more votes than any other answer; questions with tied top answers were treated as having no determinate group answer. Group-answer coverage was defined as the proportion of questions for which a voting rule produced a group answer, and group-answer accuracy was defined as the proportion of questions with a determinate group answer for which the consensus answer matched the answer key. Question difficulty was summarized according to the number of models that answered each question correctly, ranging from 0 of 6 to 6 of 6 models. Questions answered correctly by fewer models were considered more difficult, whereas questions answered correctly by more models were considered less difficult.

Observed reasoning-related interface latency was measured using a screen-recording-based timing protocol. For each question–response interaction in each run, the testing process was video-recorded. The measured interval was defined as the time from prompt submission, marked by clicking the “send” button, to completion of visible response generation, defined as the point at which the on-screen response stopped updating. Timing values were obtained by replaying the recordings and measuring the full interval for each response using a uniform protocol across all models. For latency analyses, the three run-level timing values for each model–question pair were first aggregated into a single value using the median. The resulting model–question-level timing observation was then classified according to the final model-level answer derived from the three-run majority rule.

### Statistical analysis

All statistical analyses were performed using SPSS Statistics for Windows (v. 22.0, IBM Corp) and R statistical software (v. 4.3.3). Categorical variables were summarized as counts and percentages. Accuracy estimates, group-answer coverage, and group-answer accuracy were reported with 95% CIs. Because each pair of models answered the same set of questions, pairwise comparisons of model accuracy were performed using exact McNemar tests rather than independent-sample tests. Pairwise comparisons were conducted separately for all questions, image-based questions, and nonimage-based questions. Bonferroni correction was applied within each subset to account for 15 pairwise model comparisons. Inter-model agreement for categorical answers was assessed using Cohen’s *κ*, and unadjusted percentage agreement was also reported to aid interpretation. Agreement analyses were performed separately for all questions, image-based questions, and nonimage-based questions.

To account for potential clustering of questions within OCTCases cases, a case-level bootstrap sensitivity analysis was performed. The 78 cases were resampled with replacement, and all questions belonging to each sampled case were retained. Model accuracy and the difference between nonimage-based and image-based accuracy were recalculated in each bootstrap sample. Percentile-based 95% CIs were obtained from 5,000 bootstrap iterations.

Because interface latency was an exploratory usability-related measure and timing observations may not be independent across repeated runs, questions, and cases, latency was analyzed descriptively. Available timing observations, correct and incorrect timing counts and median latency with interquartile range were summarized for each model and question subset. No inferential statistical testing was performed for latency comparisons. All statistical tests were two-sided, and a *p* < 0.05 was considered statistically significant.

## Results

### Question set characteristics

A total of 226 multiple-choice retinal questions from 78 OCTCases cases were included, comprising 151 image-based and 75 nonimage-based questions. Imaging finding or anatomy recognition was the most common question type (102/226, 45.1%), followed by diagnosis (41/226, 18.1%), other knowledge-based questions (34/226, 15.0%), treatment or management (29/226, 12.8%), staging, etiology, or risk questions (13/226, 5.8%), and investigation or testing questions (7/226, 3.1%). The question set covered a broad range of retinal topics, including other or mixed retinal conditions, vitreoretinal interface disorders or macular hole, vascular or diabetic retinal disease, age-related macular degeneration or choroidal neovascularization spectrum conditions, and other retinal disease categories (Supplementary Table S2).

### Model-level accuracy and run-level stability

Model-level accuracy based on the three-run majority rule is summarized in Table 1. Overall accuracy ranged from 59.3 to 77.4%, with the highest accuracy observed for ChatGPT-5.5 Thinking (175/226, 77.4%; 95% CI, 71.6–82.4%), followed by Gemini 3 (172/226, 76.1%; 95% CI, 70.1–81.2%) and ChatGPT-5.5 Instant (171/226, 75.7%; 95% CI, 69.7–80.8%). DeepSeek V4 showed the lowest overall accuracy (134/226, 59.3%; 95% CI, 52.8–65.5%).

**Table 1:** Performance of six LLMs across question subsets.

Model	All (%)	All 95% CI	Image-based (%)	Image-based 95% CI	Nonimage-based (%)	Nonimage-based 95% CI
ChatGPT-5.5 Instant	171/226 (75.7%)	69.7–80.8	106/151 (70.2%)	62.5–76.9	65/75 (86.7%)	77.2–92.6
ChatGPT-5.5 Thinking	175/226 (77.4%)	71.6–82.4	110/151 (72.8%)	65.3–79.3	65/75 (86.7%)	77.2–92.6
Grok 4	165/226 (73.0%)	66.9–78.4	99/151 (65.6%)	57.7–72.7	66/75 (88.0%)	78.7–93.6
Gemini 3	172/226 (76.1%)	70.1–81.2	112/151 (74.2%)	66.7–80.5	60/75 (80.0%)	69.6–87.5
DeepSeek V4	134/226 (59.3%)	52.8–65.5	75/151 (49.7%)	41.8–57.6	59/75 (78.7%)	68.1–86.4
Kimi 2.5	168/226 (74.3%)	68.3–79.6	108/151 (71.5%)	63.9–78.1	60/75 (80.0%)	69.6–87.5

CI, confidence interval; LLM, large language model.

Performance was numerically lower for image-based questions than for nonimage-based questions across all models. In the image-based subset, Gemini 3 achieved the highest accuracy (112/151, 74.2%; 95% CI, 66.7–80.5%), whereas DeepSeek V4 showed the lowest accuracy (75/151, 49.7%; 95% CI, 41.8–57.6%). In the nonimage-based subset, Grok 4 achieved the highest accuracy (66/75, 88.0%; 95% CI, 78.7–93.6%), whereas DeepSeek V4 showed the lowest accuracy (59/75, 78.7%; 95% CI, 68.1–86.4%). The case-level bootstrap sensitivity analysis showed broadly consistent performance patterns after accounting for clustering of questions within OCTCases cases (Supplementary Table S4). The nonimage-based minus image-based accuracy difference was positive for all models, although the bootstrap confidence intervals crossed zero for Gemini 3 and Kimi 2.5.

Individual-run accuracies were generally close to the three-run majority-rule accuracies (Supplementary Table S3). Across the six models, overall individual-run accuracy ranged from 58.0 to 77.9%, whereas majority-rule accuracy ranged from 59.3 to 77.4%. Across all questions, 3/3 identical answers across the three runs were observed in 57.5 to 60.6% of questions, and 2/3 identical answers were observed in 39.4 to 42.5%. No question produced three different answers across the three independent runs for any model.

Pairwise exact McNemar comparisons showed that statistically significant differences after Bonferroni correction were mainly observed in comparisons involving DeepSeek V4 in the overall and image-based subsets (Supplementary Table S5). Differences among the other models were generally not significant, and no significant pairwise differences were observed in the nonimage-based subset.

### Voting-based group-answer performance and item difficulty

Voting-based group-answer performance is shown in Table 2. Under the majority-vote rule, in which at least four of six models were required to select the same answer, a determinate group answer was generated for 182 of 226 questions, corresponding to a coverage of 80.5% (95% CI, 74.9–85.2%). Among these covered questions, 163 consensus answers were correct, yielding a consensus accuracy of 89.6% (95% CI, 84.3–93.2%). In the image-based subset, majority-vote coverage was 76.8% (116/151), and consensus accuracy was 87.1% (101/116; 95% CI, 79.8–92.0%). In the nonimage-based subset, majority-vote coverage was 88.0% (66/75), and consensus accuracy was 93.9% (62/66; 95% CI, 85.4–97.6%).

**Table 2:** Performance of group answers generated using two voting rules across six LLMs.

Subset	All models correct	All models wrong	Voting rule	Questions with group answer	Coverage, % (95% CI)	Correct consensus answers	Consensus accuracy, % (95% CI)	Tied/not covered
All questions	76	8	Majority vote (≥4/6)	182/226	80.5 (74.9–85.2)	163/182	89.6 (84.3–93.2)	44
Image-based	34	8	Majority vote (≥4/6)	116/151	76.8 (69.5–82.8)	101/116	87.1 (79.8–92.0)	35
Nonimage-based	42	0	Majority vote (≥4/6)	66/75	88.0 (78.7–93.6)	62/66	93.9 (85.4–97.6)	9
All questions	76	8	Plurality vote (unique top answer)	213/226	94.2 (90.4–96.6)	182/213	85.4 (80.1–89.6)	13
Image-based	34	8	Plurality vote (unique top answer)	141/151	93.4 (88.2–96.4)	115/141	81.6 (74.4–87.1)	10
Nonimage-based	42	0	Plurality vote (unique top answer)	72/75	96.0 (88.9–98.6)	67/72	93.1 (84.8–97.0)	3

CI, confidence interval.

Under the plurality-vote rule, in which one answer was required to receive more votes than any other answer, a determinate group answer was generated for 213 of 226 questions, corresponding to a higher coverage of 94.2% (95% CI, 90.4–96.6%). Among these covered questions, 182 consensus answers were correct, yielding a consensus accuracy of 85.4% (95% CI, 80.1–89.6%). Thus, compared with the majority-vote rule, the plurality-vote rule increased coverage but showed lower consensus accuracy among questions with a determinate group answer.

**Figure 1:**
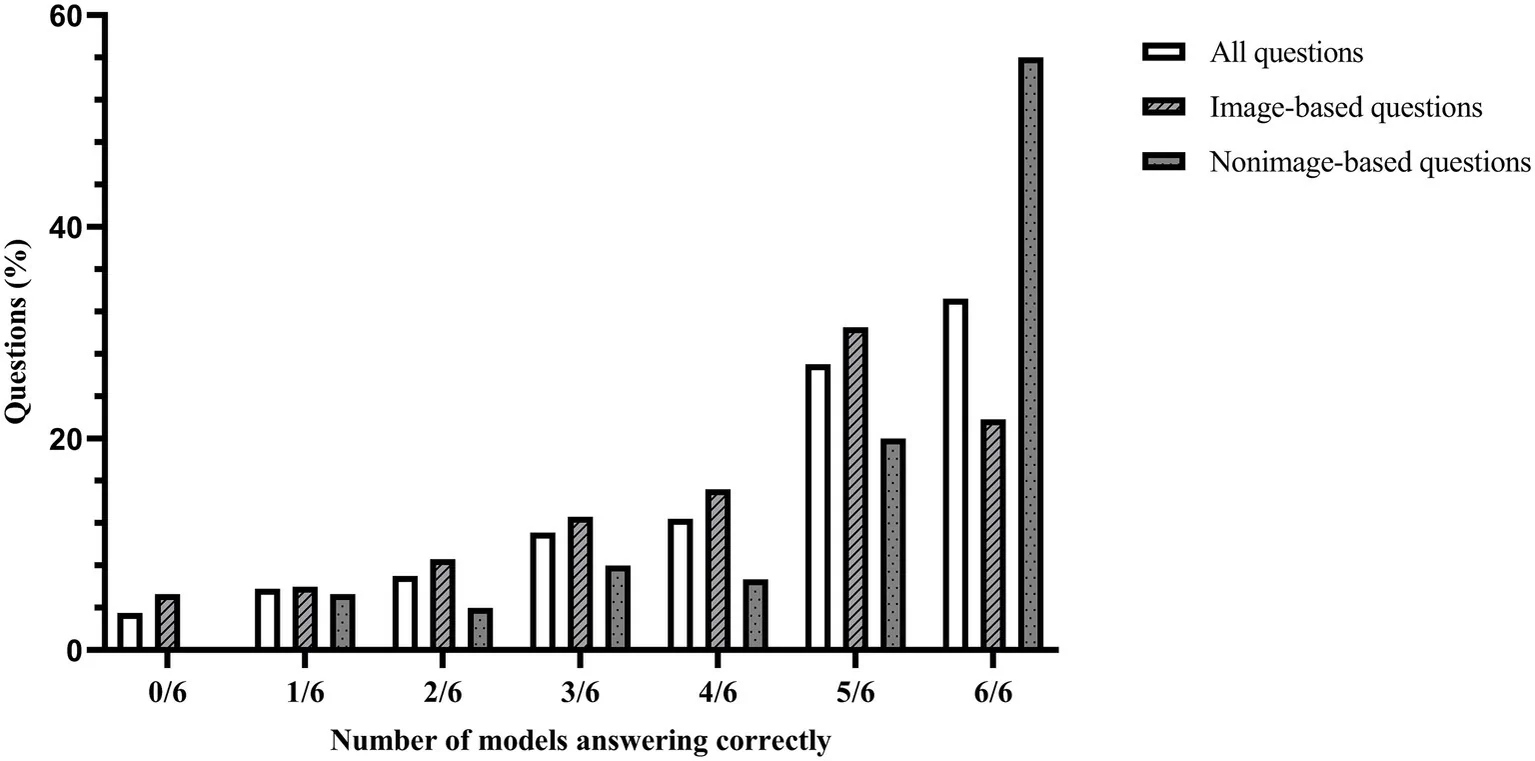
Distribution of question difficulty according to the number of models answering correctly.

Across all questions, 76 questions were answered correctly by all six models, whereas 8 questions were answered incorrectly by all six models. All unanimously incorrect questions occurred in the image-based subset. The distribution of item difficulty according to the number of models answering each question correctly is shown in Figure 1. Nonimage-based questions were more frequently answered correctly by all six models (42/75, 56.0%) than image-based questions (34/151, 22.5%), whereas image-based questions showed a broader distribution across intermediate difficulty levels.

### Inter-model agreement

Inter-model agreement is summarized in Table 3. Across all questions, the highest agreement was observed between ChatGPT-5.5 Instant and ChatGPT-5.5 Thinking, with an unadjusted agreement of 82.3% and a Cohen’s *κ* of 0.771. The same model pair also showed the highest agreement in the image-based subset (80.1%; κ = 0.739) and nonimage-based subset (86.7%; *κ* = 0.828). Agreement was generally higher for nonimage-based questions than for image-based questions across model pairs. Model pairs involving DeepSeek V4 tended to show lower agreement, particularly in the image-based subset. For example, Grok 4 versus DeepSeek V4 showed an image-based agreement of 46.4% with *κ* = 0.298, and Gemini 3 versus DeepSeek V4 showed an image-based agreement of 51.0% with κ = 0.355.

**Table 3:** Inter-model agreement for categorical answers across question subsets.

Pair	All agreement, %	All κ	Image-based agreement, %	Image-based κ	Nonimage-based agreement, %	Nonimage-based κ
ChatGPT-5.5 Instant vs. ChatGPT-5.5 Thinking	82.3	0.771	80.1	0.739	86.7	0.828
ChatGPT-5.5 Instant vs. Grok 4	70.4	0.615	64.9	0.540	81.3	0.758
ChatGPT-5.5 Instant vs. Gemini 3	67.7	0.578	61.6	0.496	80.0	0.737
ChatGPT-5.5 Instant vs. DeepSeek V4	59.7	0.476	52.3	0.376	74.7	0.665
ChatGPT-5.5 Instant vs. Kimi 2.5	68.6	0.590	64.2	0.529	77.3	0.706
ChatGPT-5.5 Thinking vs. Grok 4	70.8	0.622	64.9	0.540	82.7	0.777
ChatGPT-5.5 Thinking vs. Gemini 3	71.7	0.631	66.9	0.563	81.3	0.758
ChatGPT-5.5 Thinking vs. DeepSeek V4	61.9	0.506	55.0	0.411	76.0	0.687
ChatGPT-5.5 Thinking vs. Kimi 2.5	72.6	0.643	70.2	0.606	77.3	0.709
Grok 4 vs. Gemini 3	66.4	0.561	60.9	0.486	77.3	0.704
Grok 4 vs. DeepSeek V4	56.2	0.430	46.4	0.298	76.0	0.684
Grok 4 vs. Kimi 2.5	63.3	0.523	58.9	0.460	72.0	0.640
Gemini 3 vs. DeepSeek V4	60.6	0.483	51.0	0.355	80.0	0.734
Gemini 3 vs. Kimi 2.5	70.8	0.615	67.5	0.567	77.3	0.704
DeepSeek V4 vs. Kimi 2.5	58.4	0.459	52.3	0.374	70.7	0.618

Inter-model agreement was assessed based on the final categorical answer selected by each model after the three-run majority rule. Unadjusted percentage agreement is reported alongside Cohen’s κ.

### Interface latency and response correctness

Interface latency was analyzed as an exploratory descriptive outcome among models with available timing data (Supplementary Table S6). Timing data were not available for ChatGPT-5.5 Instant. Among the remaining models, median latency across all questions ranged from 8.0 s for DeepSeek V4 to 27.0 s for Kimi 2.5. Descriptively, ChatGPT-5.5 Thinking showed shorter median latency for correct than incorrect responses across all questions, whereas DeepSeek V4 showed longer median latency for correct than incorrect responses in the overall and image-based subsets. Gemini 3 showed nearly identical latency values for correct and incorrect responses, and Kimi 2.5 showed no consistent correct–incorrect latency pattern across subsets. These findings indicate that observed interface latency should not be interpreted as a direct proxy for response accuracy.

## Discussion

In this cross-sectional benchmark study based on retinal multiple-choice questions from OCTCases, contemporary multimodal LLMs demonstrated moderate to good performance, but their reliability varied substantially across models and question subsets. Rather than identifying a single uniformly superior model, the findings highlight several clinically relevant patterns. First, model performance was consistently lower for image-based questions than for nonimage-based questions, suggesting that retinal image interpretation remains a major limitation of current multimodal LLMs. Second, although several models showed broadly comparable overall accuracy, performance varied according to question modality, with ChatGPT-5.5 Thinking achieving the highest overall accuracy, Gemini 3 performing best on image-based questions, and Grok 4 performing best on nonimage-based questions. Third, voting-based group answers improved accuracy when sufficient model agreement was present. Fourth, inter-model agreement was lower in image-based questions, indicating greater divergence when retinal imaging interpretation was required. Finally, interface latency showed model-dependent associations with response correctness and should not be used as a direct surrogate for answer accuracy.

The performance gap between image-based and nonimage-based questions is one of the most important findings of this study. Previous evaluations of LLMs in ophthalmology have generally suggested that these models can perform well on structured knowledge-based questions, patient education tasks, and text-based clinical scenarios (9, 16, 17). However, image-centered ophthalmic tasks impose additional demands on visual recognition, anatomical localization, and multimodal reasoning (11, 18). In the present study, nonimage-based questions could often be answered using the clinical stem, answer options, and general retinal knowledge. In contrast, image-based questions required the integration of textual context with retinal imaging findings. This distinction is particularly important in retina, where subtle differences among intraretinal fluid, subretinal fluid, epiretinal membrane, macular hole, retinoschisis, pigment epithelial detachment, choroidal neovascularization, or ischemic retinal findings may substantially alter the final answer (8, 19, 20). The lower accuracy and lower inter-model agreement observed in image-based questions therefore suggest that current multimodal LLMs remain less stable in visual interpretation than in text-based medical knowledge reasoning.

Recent domain-adapted medical and ophthalmic vision-language models, including Multi-Granular Language Learning and VisionUnite, provide important context for these findings (21, 22). These studies suggest that image-centered medical tasks may benefit from specialized image-text pretraining and ophthalmology-specific clinical knowledge. Therefore, the lower and less stable performance observed for image-based retinal questions should not be interpreted as a limitation of multimodal AI in general, but rather as evidence that off-the-shelf general-purpose multimodal LLMs still require further validation and may be complemented by domain-specific ophthalmic foundation models.

The model-level findings should be interpreted with caution. Although ChatGPT-5.5 Thinking achieved the highest overall accuracy, the best-performing model differed by question subset. Gemini 3 achieved the highest accuracy in image-based questions, whereas Grok 4 performed best in nonimage-based questions. Moreover, pairwise comparisons showed that differences among the better-performing models were generally not statistically significant after correction, whereas significant differences were mainly driven by the lower performance of DeepSeek V4. These findings indicate that ranking models solely by overall accuracy may be misleading. A more informative evaluation should consider whether a model’s apparent strength lies in retinal image interpretation, text-based knowledge reasoning, or the integration of both. This is especially relevant for ophthalmology, where clinical reasoning often depends on combining patient context with high-resolution imaging features.

The individual-run analyses further showed that single-run accuracies were generally close to the three-run majority-rule accuracies, suggesting that the majority rule did not substantially inflate overall model-level performance estimates. Nevertheless, a substantial proportion of questions showed 2/3 rather than 3/3 agreement across runs, indicating that within-model response variability remains relevant even under standardized single-turn testing.

This approach is related to recent ophthalmic LLM benchmark designs. Argon and Durmuş evaluated four LLMs on cornea and external disease multiple-choice questions and considered repeated responses as well as the clinical significance of incorrect answers (23). In the present study, the three-run majority rule was used to reduce stochastic variation and derive a stable model-level answer. However, unlike that study, we did not formally classify incorrect answers according to potential clinical harm. Future retinal LLM benchmarks should incorporate harm-based error grading in addition to accuracy and response stability.

The voting-based group-answer analysis provides an additional perspective beyond single-model accuracy (24, 25). Among questions for which a determinate consensus answer was generated, voting-based consensus showed high accuracy. However, this consensus accuracy was calculated only within the subset of covered questions and should not be directly compared with individual model accuracy calculated across the full question set. The voting-based findings should therefore be interpreted as a coverage-dependent reliability signal rather than evidence that ensemble voting uniformly outperforms individual models across all questions. However, this stricter rule did not generate a group answer for all questions. By contrast, the plurality-vote rule, in which one answer received more votes than any other answer, generated group answers for more questions but with lower accuracy. This tradeoff between coverage and accuracy suggests that agreement across models may function as a practical reliability signal (26). When several models converge on the same answer, the answer is more likely to be correct; conversely, absence of a determinate group answer or tied top answers may identify questions that are difficult or visually ambiguous for current models (27). Notably, all questions answered incorrectly by all six models were image-based, highlighting a clinically relevant pattern of shared model failure in visually demanding retinal tasks. In future studies using real-world patient-level data, model disagreement or failure to generate a determinate group answer could be investigated as a potential flag for difficult or visually ambiguous retinal cases requiring specialist review (28).

Inter-model agreement further supports this interpretation. Agreement was highest between ChatGPT-5.5 Instant and ChatGPT-5.5 Thinking, which is expected given their shared model family. However, agreement generally decreased in image-based questions across model pairs, suggesting that visual interpretation increased model divergence. Model pairs involving DeepSeek V4 also tended to show lower agreement, particularly in image-based questions. These findings suggest that different multimodal LLMs may rely on different visual-textual reasoning patterns when answering retinal imaging questions (29, 30). Therefore, model disagreement should not be viewed merely as statistical noise. Instead, it may reflect meaningful uncertainty in retinal image interpretation and could serve as a practical signal for questions requiring specialist review (31).

Interface latency should also be interpreted cautiously. In this study, interface latency was measured from screen recordings as the elapsed time from prompt submission to completion of visible response generation, rather than as internal model inference time. The association between interface latency and response correctness was inconsistent across models. ChatGPT-5.5 Thinking generated correct responses faster than incorrect responses, whereas DeepSeek V4 showed longer latency for correct responses in the overall and image-based subsets. Gemini 3 and Kimi 2.5 showed no consistent correct-incorrect latency differences. These findings indicate that longer visible response generation time does not necessarily reflect more accurate reasoning. Overall, the main contribution of this study is not simply the comparison of six model accuracies, but the demonstration that image-dependent retinal questions expose greater model instability, that voting-based agreement may provide a reliability signal, that shared model failure concentrates in visually demanding questions, and that interface latency should not be interpreted as a proxy for reasoning quality (26, 31). Another important consideration is that ChatGPT-5.5 Instant and ChatGPT-5.5 Thinking belong to the same model family. Therefore, the six model votes were not fully independent, and including two related configurations may have given this model family greater weight in the consensus analysis.

Several limitations should be acknowledged. First, OCTCases is an educational case-based platform, and its multiple-choice questions may not fully represent real-world retinal diagnosis, open-ended clinical reasoning, referral decisions, or patient management. Second, the OCTCases answer key was used as the primary reference standard and was reviewed by retina specialists, but this process may not fully replace a formal independent adjudication panel, and formal inter-reviewer agreement was not calculated. Third, because OCTCases is publicly accessible, some case wording, images, explanations, or answer keys may have been included in model training data. Although web browsing and retrieval tools were not used during testing, prior training-data exposure or memorization cannot be excluded. Fourth, image-based questions included heterogeneous imaging modalities and retinal conditions, but the study was not designed to evaluate performance separately for each disease category or imaging modality. Fifth, all models were accessed through public user-facing interfaces; therefore, backend model updates, hidden system prompts, platform-specific image preprocessing, server load, regional access, and interface behavior could not be fully controlled. The results should therefore be interpreted as a time-stamped benchmark under the tested conditions. Sixth, the study used a standardized single-turn prompting framework, whereas alternative prompts, multi-turn interaction, or clinician-guided refinement may yield different results. Finally, the present study evaluated answer accuracy, model agreement, and response stability, but did not formally classify incorrect answers according to potential clinical harm. Interface latency reflected visible response completion time rather than internal model inference time and should be interpreted only as an exploratory usability-related measure.

In conclusion, contemporary multimodal LLMs showed promising but uneven performance on retinal OCTCases questions. Their performance was consistently better on nonimage-based questions than on image-based questions, and model disagreement was more prominent when retinal image interpretation was required. Voting-based group answers improved accuracy when sufficient model agreement was present, whereas absence of a determinate group answer may help identify difficult or visually ambiguous questions. These findings suggest that current multimodal LLMs may be useful for retinal education and structured question answering, but retinal imaging interpretation remains a key limitation requiring continued model development, rigorous benchmarking, and specialist oversight.

## Data Availability

The raw data supporting the conclusions of this article will be made available by the authors, without undue reservation.

## Footnotes

1
www.octcases.com
