# Supplementary material for: Comparative performance of contemporary multimodal large language models in retinal imaging question answering
Source: Front Med (Lausanne). 2026 Aug 20;13:1934968. doi: 10.3389/fmed.2026.1934968 (PMC13537951; doi:10.3389/fmed.2026.1934968)
Supplement: Supplementary file 1 [file Table_1.DOCX]

**Supplementary Methods. Prompt structure and model testing configuration**

“For each question, the prompt followed the original OCTCases structure. No additional instruction, explanation, or task-specific prompt engineering was added. The general prompt structure was as follows:

Case *[case number]*

Patient Presentation. *[Original OCTCases patient presentation and relevant clinical information]*

*[Original OCTCases imaging statement, for example: “OCT macula images were taken and shown below:”]*

Question *[question number]*: *[Original OCTCases question text]*

A. *[Option A]*
B. *[Option B]*
C. *[Option C]*
D. *[Option D]*
E. *[Option E, if available]*

For image-based questions, the corresponding retinal image files were uploaded together with the above text. For nonimage-based questions, only the text prompt was provided. The model response was recorded after the first complete answer was generated.”

For image-based questions, all retinal images associated with the corresponding OCTCases question were saved as PNG files and uploaded together with the text prompt. Images were uploaded at their original saved resolutions; image resolution was not manually adjusted or recorded. When multiple images were provided for the same question, they were uploaded in the same order as displayed on the OCTCases platform. The image order was not rearranged according to imaging modality, laterality, or perceived diagnostic importance. No image was intentionally cropped, annotated, compressed, contrast-adjusted, enhanced, or otherwise modified before upload.

| **Supplementary Table S1 Model testing configuration** | | | |
| --- | --- | --- | --- |
| Interface | Subscription level | Thinking mode | Access dates |
| ChatGPT-5.5 Instant | ChatGPT Pro | Not used; instant/default mode | 8–14 June 2026 |
| ChatGPT-5.5 Thinking | ChatGPT Pro | Thinking mode enabled | 8–14 June 2026 |
| Grok 4 | SuperGrok | Reasoning mode enabled | 8–14 June 2026 |
| Gemini 3 | Gemini Pro | Thinking/reasoning mode enabled | 8–14 June 2026 |
| DeepSeek V4 | No paid subscription tier used | Reasoning mode enabled | 8–14 June 2026 |
| Kimi 2.5 | Kimi Moderato | Thinking/reasoning mode enabled | 8–14 June 2026 |

| **Supplementary Table S2 Distribution of question characteristics in the OCTCases retinal question set** | | |
| --- | --- | --- |
| **Question type** | | |
| **Question type** | **No. of questions** | **%** |
| Imaging finding/anatomy | 102 | 45.1 |
| Diagnosis | 41 | 18.1 |
| Other knowledge | 34 | 15 |
| Treatment/management | 29 | 12.8 |
| Staging/etiology/risk | 13 | 5.8 |
| Investigation/testing | 7 | 3.1 |
| **Topic category** | | |
| Other / mixed retina | 56 | 24.8 |
| Vitreoretinal interface / macular hole | 43 | 19 |
| Vascular / diabetic retina | 43 | 19 |
| AMD / CNV spectrum | 34 | 15 |
| Retinal detachment / schisis | 22 | 9.7 |
| Inherited / toxic maculopathy | 12 | 5.3 |
| Postoperative / inflammatory CME | 5 | 2.2 |
| Inflammatory / infectious | 4 | 1.8 |
| Choroidal lesion / tumor-like | 4 | 1.8 |
| Pachychoroid / CSC / FCE | 3 | 1.3 |
| AMD = age-related macular degeneration; CNV = choroidal neovascularization; CME = cystoid macular edema; CSC = central serous chorioretinopathy; FCE = focal choroidal excavation. | | |

| **Supplementary Table S3 Individual-run accuracy and within-model response stability** | | | | | | | |
| --- | --- | --- | --- | --- | --- | --- | --- |
| **Model** | **Subset** | **Run 1** | **Run 2** | **Run 3** | **Majority rule** | **3/3 same answer** | **2/3 same answer** |
| ChatGPT-5.5 Instant | All questions | 169/226 (74.8%) | 171/226 (75.7%) | 173/226 (76.5%) | 171/226 (75.7%) | 132 (58.4%) | 94 (41.6%) |
|  | Image-based | 108/151 (71.5%) | 110/151 (72.8%) | 110/151 (72.8%) | 106/151 (70.2%) | 84 (55.6%) | 67 (44.4%) |
|  | Nonimage-based | 61/75 (81.3%) | 61/75 (81.3%) | 63/75 (84.0%) | 65/75 (86.7%) | 48 (64.0%) | 27 (36.0%) |
| ChatGPT-5.5 Thinking | All questions | 176/226 (77.9%) | 173/226 (76.5%) | 176/226 (77.9%) | 175/226 (77.4%) | 137 (60.6%) | 89 (39.4%) |
|  | Image-based | 116/151 (76.8%) | 110/151 (72.8%) | 113/151 (74.8%) | 110/151 (72.8%) | 87 (57.6%) | 64 (42.4%) |
|  | Nonimage-based | 60/75 (80.0%) | 63/75 (84.0%) | 63/75 (84.0%) | 65/75 (86.7%) | 50 (66.7%) | 25 (33.3%) |
| Grok 4 | All questions | 165/226 (73.0%) | 168/226 (74.3%) | 162/226 (71.7%) | 165/226 (73.0%) | 130 (57.5%) | 96 (42.5%) |
|  | Image-based | 101/151 (66.9%) | 103/151 (68.2%) | 103/151 (68.2%) | 99/151 (65.6%) | 80 (53.0%) | 71 (47.0%) |
|  | Nonimage-based | 64/75 (85.3%) | 65/75 (86.7%) | 59/75 (78.7%) | 66/75 (88.0%) | 50 (66.7%) | 25 (33.3%) |
| Gemini 3 | All questions | 172/226 (76.1%) | 172/226 (76.1%) | 172/226 (76.1%) | 172/226 (76.1%) | 133 (58.8%) | 93 (41.2%) |
|  | Image-based | 118/151 (78.1%) | 111/151 (73.5%) | 114/151 (75.5%) | 112/151 (74.2%) | 91 (60.3%) | 60 (39.7%) |
|  | Nonimage-based | 54/75(72.0%) | 61/75 (81.3%) | 58/75 (77.3%) | 60/75 (80.0%) | 42 (56.0%) | 33 (44.0%) |
| DeepSeek V4 | All questions | 136/226 (60.2%) | 131/226 (58.0%) | 135/226 (59.7%) | 134/226 (59.3%) | 130 (57.5%) | 96 (42.5%) |
|  | Image-based | 76/151 (50.3%) | 79/151 (52.3%) | 80/151 (53.0%) | 75/151 (49.7%) | 83 (55.0%) | 68 (45.0%) |
|  | Nonimage-based | 60/75 (80.0%) | 52/75 (69.3%) | 55/75 (73.3%) | 59/75 (78.7%) | 47 (62.7%) | 28 (37.3%) |
| Kimi 2.5 | All questions | 167/226 (73.9%) | 168/226 (74.3%) | 169/226 (74.8%) | 168/226 (74.3%) | 130 (57.5%) | 96 (42.5%) |
|  | Image-based | 105/151 (69.5%) | 112/151 (74.2%) | 107/151 (70.9%) | 108/151 (71.5%) | 81 (53.6%) | 70 (46.4%) |
|  | Nonimage-based | 62/75 (82.7%) | 56/75 (74.7%) | 62/75 (82.7%) | 60/75 (80.0%) | 49 (65.3%) | 26 (34.7%) |
| No question produced three different answers across the three independent runs for any model | | | | | | | |

| **Supplementary Table S4 Case-level bootstrap sensitivity analysis** | | | | | |
| --- | --- | --- | --- | --- | --- |
| **Model** | **Overall accuracy, % (bootstrap 95% CI)** | **Image-based accuracy, % (bootstrap 95% CI)** | **Nonimage-based accuracy, % (bootstrap 95% CI)** | **Nonimage − image difference, pp** | **Bootstrap 95% CI for difference, pp** |
| ChatGPT-5.5 Instant | 75.7 (69.6–81.1) | 70.2 (61.6–77.8) | 86.7 (77.9–94.4) | 16.5 | 4.1–28.4 |
| ChatGPT-5.5 Thinking | 77.4 (71.6–82.9) | 72.8 (64.8–80.2) | 86.7 (79.2–93.7) | 13.8 | 3.8–24.6 |
| Grok 4 | 73.0 (66.5–78.9) | 65.6 (57.4–73.4) | 88.0 (80.6–94.8) | 22.4 | 11.5–33.4 |
| Gemini 3 | 76.1 (69.5–82.4) | 74.2 (66.7–81.3) | 80.0 (69.5–90.1) | 5.8 | -5.7–17.0 |
| DeepSeek V4 | 59.3 (52.8–65.9) | 49.7 (41.1–58.2) | 78.7 (69.0–87.4) | 29.0 | 16.0–41.2 |
| Kimi 2.5 | 74.3 (67.1–81.3) | 71.5 (62.9–79.5) | 80.0 (70.4–89.0) | 8.5 | -1.9–19.2 |
| OCTCases cases were resampled with replacement, and all questions within each sampled case were retained. Differences are nonimage-based minus image-based accuracy.  CI = confidence interval. | | | | | |

| **Supplementary Table S5 Pairwise McNemar comparisons of model accuracy across question subsets** | | | | | |
| --- | --- | --- | --- | --- | --- |
| **Subset** | **Comparison** | **Model A only correct** | **Model B only correct** | **Exact McNemar p** | **Bonferroni p** |
| All questions | ChatGPT-5.5 Instant vs ChatGPT-5.5 Thinking | 15 | 19 | 0.608 | 1.000 |
|  | ChatGPT-5.5 Instant vs Grok 4 | 31 | 25 | 0.504 | 1.000 |
|  | ChatGPT-5.5 Instant vs Gemini 3 | 33 | 34 | 1.000 | 1.000 |
|  | ChatGPT-5.5 Instant vs DeepSeek V4 | 56 | 19 | <0.001 | <0.001 |
|  | ChatGPT-5.5 Instant vs Kimi 2.5 | 31 | 28 | 0.795 | 1.000 |
|  | ChatGPT-5.5 Thinking vs Grok 4 | 31 | 21 | 0.212 | 1.000 |
|  | ChatGPT-5.5 Thinking vs Gemini 3 | 28 | 25 | 0.784 | 1.000 |
|  | ChatGPT-5.5 Thinking vs DeepSeek V4 | 54 | 13 | <0.001 | <0.001 |
|  | ChatGPT-5.5 Thinking vs Kimi 2.5 | 30 | 23 | 0.410 | 1.000 |
|  | Grok 4 vs Gemini 3 | 30 | 37 | 0.464 | 1.000 |
|  | Grok 4 vs DeepSeek V4 | 56 | 25 | <0.001 | 0.011 |
|  | Grok 4 vs Kimi 2.5 | 34 | 37 | 0.813 | 1.000 |
|  | Gemini 3 vs DeepSeek V4 | 57 | 19 | <0.001 | <0.001 |
|  | Gemini 3 vs Kimi 2.5 | 28 | 24 | 0.678 | 1.000 |
|  | DeepSeek V4 vs Kimi 2.5 | 21 | 55 | <0.001 | 0.002 |
| Image-based | ChatGPT-5.5 Instant vs ChatGPT-5.5 Thinking | 10 | 14 | 0.541 | 1.000 |
|  | ChatGPT-5.5 Instant vs Grok 4 | 25 | 18 | 0.360 | 1.000 |
|  | ChatGPT-5.5 Instant vs Gemini 3 | 24 | 30 | 0.497 | 1.000 |
|  | ChatGPT-5.5 Instant vs DeepSeek V4 | 44 | 13 | <0.001 | <0.001 |
|  | ChatGPT-5.5 Instant vs Kimi 2.5 | 21 | 23 | 0.880 | 1.000 |
|  | ChatGPT-5.5 Thinking vs Grok 4 | 26 | 15 | 0.117 | 1.000 |
|  | ChatGPT-5.5 Thinking vs Gemini 3 | 20 | 22 | 0.878 | 1.000 |
|  | ChatGPT-5.5 Thinking vs DeepSeek V4 | 43 | 8 | < 0.001 | < 0.001 |
|  | ChatGPT-5.5 Thinking vs Kimi 2.5 | 20 | 18 | 0.871 | 1.000 |
|  | Grok 4 vs Gemini 3 | 20 | 33 | 0.098 | 1.000 |
|  | Grok 4 vs DeepSeek V4 | 45 | 21 | 0.004 | 0.064 |
|  | Grok 4 vs Kimi 2.5 | 21 | 30 | 0.262 | 1.000 |
|  | Gemini 3 vs DeepSeek V4 | 50 | 13 | < 0.001 | < 0.001 |
|  | Gemini 3 vs Kimi 2.5 | 22 | 18 | 0.636 | 1.000 |
|  | DeepSeek V4 vs Kimi 2.5 | 13 | 46 | < 0.001 | < 0.001 |
| Nonimage-based | ChatGPT-5.5 Instant vs ChatGPT-5.5 Thinking | 5 | 5 | 1.000 | 1.000 |
|  | ChatGPT-5.5 Instant vs Grok 4 | 6 | 7 | 1.000 | 1.000 |
|  | ChatGPT-5.5 Instant vs Gemini 3 | 9 | 4 | 0.267 | 1.000 |
|  | ChatGPT-5.5 Instant vs DeepSeek V4 | 12 | 6 | 0.238 | 1.000 |
|  | ChatGPT-5.5 Instant vs Kimi 2.5 | 10 | 5 | 0.302 | 1.000 |
|  | ChatGPT-5.5 Thinking vs Grok 4 | 5 | 6 | 1.000 | 1.000 |
|  | ChatGPT-5.5 Thinking vs Gemini 3 | 8 | 3 | 0.227 | 1.000 |
|  | ChatGPT-5.5 Thinking vs DeepSeek V4 | 11 | 5 | 0.210 | 1.000 |
|  | ChatGPT-5.5 Thinking vs Kimi 2.5 | 10 | 5 | 0.302 | 1.000 |
|  | Grok 4 vs Gemini 3 | 10 | 4 | 0.180 | 1.000 |
|  | Grok 4 vs DeepSeek V4 | 11 | 4 | 0.118 | 1.000 |
|  | Grok 4 vs Kimi 2.5 | 13 | 7 | 0.263 | 1.000 |
|  | Gemini 3 vs DeepSeek V4 | 7 | 6 | 1.000 | 1.000 |
|  | Gemini 3 vs Kimi 2.5 | 6 | 6 | 1.000 | 1.000 |
|  | DeepSeek V4 vs Kimi 2.5 | 8 | 9 | 1.000 | 1.000 |
| “Model A only correct” indicates the number of questions answered correctly by the first model and incorrectly by the second model in each comparison; “Model B only correct” indicates the reverse discordant pattern. Bonferroni-adjusted p values account for 15 comparisons within each subset. | | | | | |

| **Supplementary Table S6 Descriptive interface latency analysis** | | | | | | | |
| --- | --- | --- | --- | --- | --- | --- | --- |
| **Model** | **Subset** | **Available timing, n** | **Correct timing, n** | **Incorrect timing, n** | **All time, median (IQR), s** | **Correct time, median (IQR), s** | **Incorrect time, median (IQR), s** |
| ChatGPT-5.5 Instant | All questions | 0 | 0 | 0 | NA | NA | NA |
|  | Image-based | 0 | 0 | 0 | NA | NA | NA |
|  | Nonimage-based | 0 | 0 | 0 | NA | NA | NA |
| ChatGPT-5.5 Thinking | All questions | 226 | 175 | 51 | 14.0 (8.0–27.0) | 12.0 (7.0–22.0) | 23.0 (13.5–42.5) |
|  | Image-based | 151 | 110 | 41 | 17.0 (9.0–34.0) | 16.0 (9.0–28.5) | 25.0 (16.0–46.0) |
|  | Nonimage-based | 75 | 65 | 10 | 9.0 (6.0–15.0) | 9.0 (6.0–13.0) | 16.5 (9.8–24.5) |
| Grok 4 | All questions | 226 | 165 | 61 | 23.0 (17.0–39.0) | 22.0 (17.0–36.0) | 25.0 (18.0–42.0) |
|  | Image-based | 151 | 99 | 52 | 23.0 (17.5–41.5) | 23.0 (17.0–42.0) | 24.0 (18.0–37.0) |
|  | Nonimage-based | 75 | 66 | 9 | 22.0 (16.5–33.5) | 22.0 (16.0–31.0) | 40.0 (27.0–59.0) |
| Gemini 3 | All questions | 226 | 172 | 54 | 13.0 (13.0–13.0) | 13.0 (13.0–13.0) | 13.0 (13.0–13.0) |
|  | Image-based | 151 | 112 | 39 | 13.0 (13.0–13.0) | 13.0 (13.0–13.0) | 13.0 (13.0–13.0) |
|  | Nonimage-based | 75 | 60 | 15 | 13.0 (13.0–13.0) | 13.0 (13.0–13.0) | 13.0 (11.5–13.0) |
| DeepSeek V4 | All questions | 226 | 134 | 92 | 8.0 (6.0–8.0) | 8.0 (6.0–10.0) | 6.0 (6.0–8.0) |
|  | Image-based | 151 | 75 | 76 | 6.0 (6.0–8.0) | 8.0 (6.0–10.0) | 6.0 (6.0–8.0) |
|  | Nonimage-based | 75 | 59 | 16 | 8.0 (6.0–8.0) | 8.0 (6.0–8.0) | 6.0 (6.0–8.5) |
| Kimi 2.5 | All questions | 226 | 168 | 58 | 27.0 (18.0–38.0) | 28.0 (19.0–38.2) | 21.5 (12.0–34.8) |
|  | Image-based | 151 | 108 | 43 | 27.0 (17.5–40.5) | 27.0 (18.8–40.2) | 23.0 (13.0–40.0) |
|  | Nonimage-based | 75 | 60 | 15 | 28.0 (18.5–37.0) | 28.0 (20.0–37.0) | 20.0 (10.0–31.5) |
| IQR = interquartile range; NA = not available. | | | | | | | |
