# Supplementary material for: Comparative performance of contemporary multimodal large language models in retinal imaging question answering
Source: Front Med (Lausanne). 2026 Aug 20;13:1934968. doi: 10.3389/fmed.2026.1934968 (PMC13537951; doi:10.3389/fmed.2026.1934968)
Supplement: Supplementary file 2 [file Table_2.DOCX]

| **Supplementary Table S7. Case/question identifiers, image-based classification, reference answers, and final model-level responses** | | | | | | | | | |
| --- | --- | --- | --- | --- | --- | --- | --- | --- | --- |
| **Case ID** | **Question ID** | **Subset** | **Reference** | **ChatGPT-5.5 Instant** | **ChatGPT-5.5 Thinking** | **Grok 4** | **Gemini 3** | **DeepSeek V4** | **Kimi 2.5** |
| 1 | 1.1 | Image-based | D | D/1 | D/1 | D/1 | C/0 | D/1 | D/1 |
| 1 | 1.2 | Nonimage-based | D | D/1 | D/1 | D/1 | D/1 | D/1 | D/1 |
| 2 | 2.1 | Image-based | B | C/0 | C/0 | B/1 | B/1 | B/1 | B/1 |
| 3 | 3.1 | Image-based | C | D/0 | D/0 | C/1 | B/0 | B/0 | B/0 |
| 3 | 3.2 | Image-based | B | D/0 | D/0 | C/0 | B/1 | B/1 | D/0 |
| 4 | 4.1 | Image-based | D | B/0 | D/1 | C/0 | D/1 | D/1 | D/1 |
| 5 | 5.1 | Image-based | D | D/1 | D/1 | D/1 | D/1 | B/0 | D/1 |
| 5 | 5.2 | Image-based | C | B/0 | A/0 | A/0 | C/1 | B/0 | C/1 |
| 5 | 5.3 | Image-based | D | D/1 | B/0 | A/0 | A/0 | D/1 | D/1 |
| 6 | 6.1 | Image-based | B | B/1 | B/1 | B/1 | D/0 | D/0 | C/0 |
| 6 | 6.2 | Nonimage-based | D | D/1 | C/0 | D/1 | D/1 | D/1 | C/0 |
| 7 | 7.1 | Image-based | A | A/1 | A/1 | A/1 | A/1 | A/1 | A/1 |
| 7 | 7.2 | Nonimage-based | D | D/1 | D/1 | D/1 | D/1 | D/1 | D/1 |
| 8 | 8.1 | Image-based | D | C/0 | C/0 | C/0 | D/1 | B/0 | D/1 |
| 8 | 8.2 | Nonimage-based | C | C/1 | C/1 | C/1 | C/1 | C/1 | C/1 |
| 9 | 9.1 | Image-based | D | A/0 | A/0 | C/0 | D/1 | C/0 | C/0 |
| 9 | 9.2 | Nonimage-based | C | A/0 | A/0 | B/0 | A/0 | C/1 | C/1 |
| 10 | 10.1 | Image-based | B | D/0 | B/1 | C/0 | D/0 | D/0 | B/1 |
| 10 | 10.2 | Image-based | B | B/1 | B/1 | D/0 | B/1 | B/1 | B/1 |
| 11 | 11.1 | Image-based | B | B/1 | B/1 | C/0 | D/0 | B/1 | B/1 |
| 11 | 11.2 | Image-based | D | D/1 | D/1 | D/1 | D/1 | D/1 | D/1 |
| 11 | 11.3 | Nonimage-based | D | D/1 | D/1 | D/1 | D/1 | D/1 | D/1 |
| 12 | 12.1 | Image-based | B | C/0 | C/0 | B/1 | B/1 | C/0 | C/0 |
| 12 | 12.2 | Image-based | B | B/1 | B/1 | B/1 | D/0 | D/0 | B/1 |
| 12 | 12.3 | Nonimage-based | C | C/1 | C/1 | C/1 | A/0 | A/0 | B/0 |
| 13 | 13.1 | Image-based | D | D/1 | D/1 | D/1 | D/1 | D/1 | B/0 |
| 13 | 13.2 | Image-based | B | B/1 | B/1 | B/1 | B/1 | B/1 | B/1 |
| 13 | 13.3 | Nonimage-based | A | A/1 | A/1 | A/1 | A/1 | A/1 | A/1 |
| 14 | 14.1 | Image-based | A | A/1 | A/1 | A/1 | A/1 | A/1 | A/1 |
| 14 | 14.2 | Image-based | B | B/1 | B/1 | B/1 | B/1 | B/1 | B/1 |
| 14 | 14.3 | Image-based | E | E/1 | D/0 | D/0 | D/0 | B/0 | E/1 |
| 14 | 14.4 | Nonimage-based | C | C/1 | C/1 | C/1 | D/0 | C/1 | D/0 |
| 14 | 14.5 | Nonimage-based | D | D/1 | C/0 | D/1 | D/1 | D/1 | D/1 |
| 15 | 15.1 | Nonimage-based | B | B/1 | B/1 | B/1 | B/1 | D/0 | B/1 |
| 16 | 16.1 | Image-based | A | D/0 | A/1 | A/1 | D/0 | D/0 | D/0 |
| 16 | 16.2 | Image-based | D | D/1 | D/1 | D/1 | D/1 | D/1 | D/1 |
| 17 | 17.1 | Nonimage-based | D | D/1 | D/1 | D/1 | D/1 | D/1 | D/1 |
| 18 | 18.1 | Image-based | A | C/0 | B/0 | C/0 | A/1 | D/0 | B/0 |
| 19 | 19.1 | Image-based | B | D/0 | D/0 | D/0 | B/1 | D/0 | D/0 |
| 19 | 19.2 | Nonimage-based | A | A/1 | A/1 | A/1 | A/1 | A/1 | A/1 |
| 20 | 20.1 | Nonimage-based | C | C/1 | C/1 | C/1 | C/1 | A/0 | C/1 |
| 20 | 20.2 | Image-based | A | A/1 | A/1 | A/1 | A/1 | A/1 | A/1 |
| 21 | 21.1 | Image-based | D | B/0 | B/0 | A/0 | D/1 | A/0 | D/1 |
| 21 | 21.2 | Image-based | D | D/1 | D/1 | B/0 | B/0 | A/0 | A/0 |
| 22 | 22.1 | Image-based | C | C/1 | C/1 | B/0 | C/1 | A/0 | A/0 |
| 22 | 22.2 | Image-based | B | B/1 | B/1 | B/1 | B/1 | B/1 | B/1 |
| 22 | 22.3 | Image-based | B | B/1 | B/1 | D/0 | B/1 | B/1 | B/1 |
| 23 | 23.1 | Image-based | C | C/1 | C/1 | C/1 | C/1 | B/0 | C/1 |
| 23 | 23.2 | Image-based | C | B/0 | C/1 | B/0 | C/1 | A/0 | C/1 |
| 23 | 23.3 | Image-based | A | A/1 | A/1 | C/0 | A/1 | A/1 | A/1 |
| 24 | 24.1 | Image-based | D | D/1 | A/0 | D/1 | D/1 | B/0 | D/1 |
| 24 | 24.2 | Nonimage-based | E | E/1 | E/1 | E/1 | E/1 | E/1 | E/1 |
| 24 | 24.3 | Image-based | A | B/0 | B/0 | C/0 | A/1 | B/0 | B/0 |
| 25 | 25.1 | Image-based | A | A/1 | A/1 | A/1 | A/1 | B/0 | A/1 |
| 25 | 25.2 | Nonimage-based | E | E/1 | E/1 | E/1 | E/1 | E/1 | E/1 |
| 25 | 25.3 | Nonimage-based | A | A/1 | A/1 | A/1 | A/1 | A/1 | A/1 |
| 25 | 25.4 | Nonimage-based | E | E/1 | E/1 | E/1 | E/1 | E/1 | E/1 |
| 26 | 26.1 | Image-based | A | A/1 | A/1 | D/0 | A/1 | A/1 | A/1 |
| 26 | 26.2 | Nonimage-based | C | C/1 | C/1 | C/1 | C/1 | C/1 | C/1 |
| 26 | 26.3 | Image-based | B | B/1 | B/1 | B/1 | B/1 | C/0 | B/1 |
| 26 | 26.4 | Image-based | C | C/1 | C/1 | B/0 | B/0 | C/1 | C/1 |
| 26 | 26.5 | Image-based | B | B/1 | B/1 | B/1 | B/1 | C/0 | B/1 |
| 27 | 27 | Nonimage-based | D | D/1 | D/1 | C/0 | D/1 | D/1 | D/1 |
| 28 | 28.1 | Image-based | D | B/0 | D/1 | D/1 | D/1 | D/1 | D/1 |
| 28 | 28.2 | Image-based | A | B/0 | B/0 | A/1 | A/1 | B/0 | A/1 |
| 28 | 28.3 | Nonimage-based | D | D/1 | D/1 | D/1 | D/1 | D/1 | D/1 |
| 29 | 29 | Nonimage-based | B | B/1 | B/1 | B/1 | B/1 | B/1 | B/1 |
| 30 | 30.1 | Image-based | B | B/1 | B/1 | B/1 | B/1 | B/1 | B/1 |
| 30 | 30.2 | Nonimage-based | E | A/0 | A/0 | E/1 | A/0 | A/0 | D/0 |
| 30 | 30.3 | Image-based | A | A/1 | A/1 | B/0 | A/1 | A/1 | B/0 |
| 31 | 31.1 | Image-based | C | A/0 | D/0 | C/1 | A/0 | A/0 | C/1 |
| 31 | 31.2 | Nonimage-based | C | C/1 | C/1 | C/1 | C/1 | C/1 | C/1 |
| 32 | 32.1 | Image-based | D | A/0 | A/0 | C/0 | B/0 | C/0 | B/0 |
| 32 | 32.2 | Nonimage-based | D | B/0 | B/0 | D/1 | B/0 | D/1 | B/0 |
| 32 | 32.3 | Image-based | D | D/1 | D/1 | D/1 | D/1 | D/1 | D/1 |
| 33 | 33 | Image-based | B | B/1 | B/1 | B/1 | B/1 | B/1 | B/1 |
| 34 | 34.1 | Image-based | B | D/0 | D/0 | D/0 | B/1 | D/0 | D/0 |
| 34 | 34.2 | Image-based | B | D/0 | B/1 | D/0 | B/1 | D/0 | B/1 |
| 35 | 35.1 | Image-based | A | B/0 | B/0 | A/1 | A/1 | D/0 | A/1 |
| 35 | 35.2 | Image-based | D | D/1 | C/0 | D/1 | C/0 | C/0 | B/0 |
| 35 | 35.3 | Nonimage-based | C | C/1 | C/1 | C/1 | C/1 | C/1 | C/1 |
| 35 | 35.4 | Nonimage-based | E | A/0 | E/1 | A/0 | E/1 | C/0 | E/1 |
| 36 | 36.1 | Image-based | B | A/0 | A/0 | A/0 | A/0 | C/0 | A/0 |
| 36 | 36.2 | Image-based | E | E/1 | E/1 | A/0 | A/0 | E/1 | E/1 |
| 36 | 36.3 | Image-based | D | D/1 | D/1 | D/1 | D/1 | D/1 | D/1 |
| 37 | 37.1 | Image-based | C | C/1 | C/1 | C/1 | C/1 | D/0 | C/1 |
| 37 | 37.2 | Image-based | B | B/1 | B/1 | B/1 | B/1 | B/1 | B/1 |
| 37 | 37.3 | Nonimage-based | D | D/1 | D/1 | D/1 | D/1 | D/1 | D/1 |
| 38 | 38.1 | Image-based | B | B/1 | B/1 | C/0 | B/1 | B/1 | B/1 |
| 38 | 38.2 | Image-based | D | C/0 | A/0 | C/0 | A/0 | A/0 | A/0 |
| 39 | 39.1 | Image-based | C | D/0 | D/0 | D/0 | C/1 | A/0 | D/0 |
| 39 | 39.2 | Nonimage-based | A | A/1 | A/1 | A/1 | A/1 | A/1 | B/0 |
| 40 | 40.1 | Image-based | A | A/1 | A/1 | A/1 | A/1 | A/1 | A/1 |
| 40 | 40.2 | Image-based | E | E/1 | E/1 | E/1 | E/1 | E/1 | E/1 |
| 40 | 40.3 | Image-based | B | B/1 | B/1 | B/1 | B/1 | B/1 | B/1 |
| 40 | 40.4 | Nonimage-based | D | D/1 | D/1 | D/1 | D/1 | D/1 | D/1 |
| 41 | 41.1 | Image-based | A | A/1 | A/1 | A/1 | A/1 | D/0 | A/1 |
| 41 | 41.2 | Image-based | A | A/1 | A/1 | A/1 | A/1 | A/1 | A/1 |
| 41 | 41.3 | Nonimage-based | D | D/1 | D/1 | D/1 | D/1 | D/1 | D/1 |
| 42 | 42 | Image-based | D | D/1 | D/1 | D/1 | D/1 | D/1 | D/1 |
| 43 | 43.1 | Image-based | A | A/1 | A/1 | A/1 | A/1 | D/0 | A/1 |
| 43 | 43.2 | Nonimage-based | B | B/1 | B/1 | B/1 | B/1 | B/1 | B/1 |
| 43 | 43.3 | Image-based | D | D/1 | D/1 | D/1 | D/1 | B/0 | D/1 |
| 43 | 43.4 | Nonimage-based | B | B/1 | B/1 | B/1 | B/1 | B/1 | B/1 |
| 44 | 44.1 | Image-based | B | D/0 | B/1 | B/1 | B/1 | B/1 | B/1 |
| 44 | 44.2 | Nonimage-based | A | A/1 | A/1 | A/1 | A/1 | A/1 | A/1 |
| 44 | 44.3 | Image-based | D | D/1 | D/1 | D/1 | D/1 | A/0 | D/1 |
| 44 | 44.4 | Nonimage-based | D | D/1 | D/1 | A/0 | D/1 | D/1 | A/0 |
| 45 | 45.1 | Image-based | C | D/0 | D/0 | D/0 | D/0 | D/0 | B/0 |
| 45 | 45.2 | Image-based | B | C/0 | B/1 | D/0 | B/1 | D/0 | B/1 |
| 45 | 45.3 | Nonimage-based | A | A/1 | A/1 | A/1 | A/1 | A/1 | A/1 |
| 46 | 46.1 | Image-based | B | B/1 | B/1 | B/1 | B/1 | A/0 | B/1 |
| 46 | 46.2 | Image-based | A | B/0 | A/1 | A/1 | A/1 | A/1 | A/1 |
| 46 | 46.3 | Nonimage-based | B | B/1 | B/1 | B/1 | B/1 | A/0 | B/1 |
| 46 | 46.4 | Nonimage-based | A | A/1 | A/1 | A/1 | A/1 | A/1 | A/1 |
| 47 | 47.1 | Nonimage-based | B | B/1 | D/0 | B/1 | B/1 | B/1 | B/1 |
| 47 | 47.2 | Nonimage-based | D | D/1 | D/1 | D/1 | D/1 | D/1 | D/1 |
| 47 | 47.3 | Nonimage-based | D | D/1 | D/1 | D/1 | D/1 | D/1 | D/1 |
| 48 | 48.1 | Nonimage-based | A | A/1 | A/1 | A/1 | A/1 | D/0 | A/1 |
| 48 | 48.2 | Image-based | A | B/0 | B/0 | B/0 | A/1 | A/1 | B/0 |
| 48 | 48.3 | Image-based | A | D/0 | A/1 | A/1 | A/1 | A/1 | A/1 |
| 48 | 48.4 | Nonimage-based | E | E/1 | E/1 | E/1 | E/1 | E/1 | E/1 |
| 49 | 49.1 | Image-based | A | A/1 | A/1 | A/1 | A/1 | E/0 | A/1 |
| 49 | 49.2 | Image-based | B | B/1 | B/1 | B/1 | B/1 | E/0 | B/1 |
| 49 | 49.3 | Nonimage-based | D | D/1 | D/1 | D/1 | D/1 | D/1 | D/1 |
| 49 | 49.4 | Nonimage-based | A | E/0 | A/1 | A/1 | A/1 | A/1 | A/1 |
| 50 | 50.1 | Image-based | C | A/0 | A/0 | C/1 | C/1 | A/0 | C/1 |
| 50 | 50.2 | Image-based | B | B/1 | B/1 | B/1 | B/1 | B/1 | B/1 |
| 51 | 51.1 | Image-based | F | C/0 | C/0 | F/1 | C/0 | B/0 | D/0 |
| 51 | 51.2 | Image-based | C | C/1 | C/1 | D/0 | C/1 | D/0 | A/0 |
| 51 | 51.3 | Nonimage-based | C | B/0 | C/1 | C/1 | C/1 | C/1 | B/0 |
| 51 | 51.4 | Image-based | E | E/1 | E/1 | E/1 | E/1 | D/0 | D/0 |
| 52 | 52.1 | Image-based | D | D/1 | A/0 | B/0 | D/1 | D/1 | D/1 |
| 52 | 52.2 | Nonimage-based | C | A/0 | C/1 | C/1 | C/1 | C/1 | C/1 |
| 52 | 52.3 | Image-based | D | D/1 | C/0 | D/1 | D/1 | C/0 | D/1 |
| 52 | 52.4 | Nonimage-based | F | F/1 | F/1 | F/1 | E/0 | E/0 | A/0 |
| 53 | 53.1 | Image-based | A | A/1 | A/1 | A/1 | A/1 | E/0 | A/1 |
| 53 | 53.2 | Image-based | B | B/1 | B/1 | B/1 | B/1 | B/1 | B/1 |
| 53 | 53.3 | Nonimage-based | D | D/1 | D/1 | D/1 | D/1 | D/1 | D/1 |
| 54 | 54.1 | Image-based | A | C/0 | C/0 | D/0 | A/1 | A/1 | A/1 |
| 54 | 54.2 | Nonimage-based | D | D/1 | D/1 | D/1 | D/1 | D/1 | D/1 |
| 54 | 54.3 | Image-based | D | D/1 | D/1 | D/1 | D/1 | D/1 | D/1 |
| 55 | 55.1 | Image-based | D | C/0 | C/0 | C/0 | C/0 | C/0 | C/0 |
| 55 | 55.2 | Image-based | B | C/0 | B/1 | B/1 | B/1 | C/0 | B/1 |
| 55 | 55.3 | Image-based | B | C/0 | D/0 | B/1 | B/1 | B/1 | A/0 |
| 55 | 55.4 | Nonimage-based | A | A/1 | A/1 | A/1 | A/1 | A/1 | A/1 |
| 55 | 55.5 | Nonimage-based | A | A/1 | A/1 | A/1 | A/1 | A/1 | A/1 |
| 56 | 56.1 | Image-based | B | B/1 | B/1 | B/1 | B/1 | A/0 | B/1 |
| 56 | 56.2 | Image-based | C | C/1 | C/1 | D/0 | C/1 | C/1 | C/1 |
| 56 | 56.3 | Image-based | D | D/1 | D/1 | B/0 | D/1 | D/1 | D/1 |
| 57 | 57.1 | Image-based | D | D/1 | D/1 | D/1 | D/1 | D/1 | D/1 |
| 57 | 57.2 | Nonimage-based | D | D/1 | D/1 | D/1 | D/1 | D/1 | D/1 |
| 57 | 57.3 | Nonimage-based | C | C/1 | C/1 | C/1 | C/1 | C/1 | C/1 |
| 57 | 57.4 | Nonimage-based | B | B/1 | B/1 | B/1 | B/1 | B/1 | B/1 |
| 58 | 58.1 | Image-based | E | E/1 | C/0 | E/1 | A/0 | B/0 | E/1 |
| 58 | 58.2 | Image-based | A | A/1 | A/1 | A/1 | A/1 | A/1 | A/1 |
| 58 | 58.3 | Nonimage-based | C | C/1 | C/1 | C/1 | C/1 | A/0 | C/1 |
| 59 | 59.1 | Image-based | B | B/1 | B/1 | A/0 | B/1 | A/0 | B/1 |
| 59 | 59.2 | Nonimage-based | D | D/1 | D/1 | D/1 | D/1 | D/1 | D/1 |
| 59 | 59.3 | Image-based | C | D/0 | D/0 | D/0 | D/0 | D/0 | D/0 |
| 60 | 60.1 | Image-based | D | D/1 | D/1 | D/1 | D/1 | D/1 | D/1 |
| 60 | 60.2 | Image-based | D | D/1 | D/1 | D/1 | D/1 | D/1 | D/1 |
| 60 | 60.3 | Image-based | A | A/1 | A/1 | A/1 | A/1 | A/1 | A/1 |
| 60 | 60.4 | Image-based | B | B/1 | B/1 | B/1 | B/1 | B/1 | B/1 |
| 61 | 61.1 | Image-based | D | D/1 | D/1 | D/1 | D/1 | B/0 | D/1 |
| 61 | 61.2 | Image-based | C | C/1 | C/1 | C/1 | A/0 | C/1 | C/1 |
| 61 | 61.3 | Image-based | D | D/1 | C/0 | A/0 | A/0 | C/0 | C/0 |
| 62 | 62.1 | Image-based | D | D/1 | D/1 | D/1 | D/1 | C/0 | D/1 |
| 62 | 62.2 | Image-based | A | C/0 | A/1 | A/1 | B/0 | A/1 | A/1 |
| 62 | 62.3 | Image-based | D | D/1 | D/1 | D/1 | D/1 | C/0 | D/1 |
| 62 | 62.4 | Nonimage-based | A | A/1 | A/1 | B/0 | A/1 | A/1 | A/1 |
| 63 | 63.1 | Image-based | H | A/0 | E/0 | A/0 | A/0 | A/0 | E/0 |
| 63 | 63.2 | Nonimage-based | B | B/1 | B/1 | A/0 | A/0 | A/0 | B/1 |
| 63 | 63.3 | Image-based | D | D/1 | D/1 | D/1 | A/0 | D/1 | D/1 |
| 63 | 63.4 | Nonimage-based | A | A/1 | A/1 | A/1 | A/1 | A/1 | A/1 |
| 63 | 63.5 | Image-based | D | D/1 | A/0 | A/0 | A/0 | A/0 | A/0 |
| 64 | 64.1 | Image-based | B | B/1 | B/1 | B/1 | B/1 | A/0 | B/1 |
| 64 | 64.2 | Nonimage-based | A | A/1 | A/1 | A/1 | B/0 | A/1 | A/1 |
| 64 | 64.3 | Nonimage-based | D | D/1 | D/1 | D/1 | B/0 | D/1 | D/1 |
| 65 | 65.1 | Image-based | E | E/1 | E/1 | E/1 | A/0 | D/0 | E/1 |
| 65 | 65.2 | Image-based | C | C/1 | C/1 | B/0 | A/0 | C/1 | C/1 |
| 65 | 65.3 | Image-based | B | D/0 | D/0 | D/0 | B/1 | C/0 | B/1 |
| 65 | 65.4 | Image-based | A | A/1 | A/1 | A/1 | A/1 | C/0 | A/1 |
| 66 | 66.1 | Image-based | A | A/1 | A/1 | C/0 | E/0 | C/0 | A/1 |
| 66 | 66.2 | Image-based | D | D/1 | C/0 | D/1 | E/0 | C/0 | D/1 |
| 66 | 66.3 | Nonimage-based | A | A/1 | B/0 | D/0 | D/0 | C/0 | A/1 |
| 66 | 66.4 | Image-based | A | B/0 | B/0 | A/1 | B/0 | A/1 | A/1 |
| 67 | 67.1 | Image-based | A | A/1 | A/1 | A/1 | A/1 | A/1 | B/0 |
| 67 | 67.2 | Nonimage-based | D | D/1 | B/0 | B/0 | A/0 | A/0 | C/0 |
| 67 | 67.3 | Nonimage-based | E | E/1 | E/1 | E/1 | A/0 | A/0 | D/0 |
| 68 | 68.1 | Nonimage-based | A | A/1 | A/1 | A/1 | A/1 | A/1 | A/1 |
| 68 | 68.2 | Image-based | C | C/1 | C/1 | C/1 | C/1 | C/1 | C/1 |
| 68 | 68.3 | Nonimage-based | A | B/0 | B/0 | A/1 | B/0 | B/0 | B/0 |
| 68 | 68.4 | Nonimage-based | C | C/1 | C/1 | C/1 | C/1 | C/1 | C/1 |
| 69 | 69.1 | Image-based | C | C/1 | C/1 | C/1 | A/0 | C/1 | A/0 |
| 69 | 69.2 | Image-based | B | B/1 | B/1 | B/1 | B/1 | D/0 | B/1 |
| 69 | 69.3 | Nonimage-based | A | A/1 | A/1 | A/1 | A/1 | A/1 | A/1 |
| 69 | 69.4 | Nonimage-based | A | A/1 | A/1 | A/1 | A/1 | A/1 | A/1 |
| 70 | 70.1 | Image-based | D | D/1 | D/1 | D/1 | D/1 | D/1 | D/1 |
| 70 | 70.2 | Image-based | A | A/1 | A/1 | A/1 | A/1 | A/1 | A/1 |
| 70 | 70.3 | Image-based | C | C/1 | C/1 | C/1 | C/1 | C/1 | C/1 |
| 70 | 70.4 | Image-based | B | B/1 | B/1 | C/0 | B/1 | B/1 | B/1 |
| 70 | 70.5 | Image-based | C | C/1 | C/1 | C/1 | C/1 | C/1 | C/1 |
| 71 | 71.1 | Image-based | C | C/1 | C/1 | C/1 | C/1 | C/1 | D/0 |
| 71 | 71.2 | Image-based | C | C/1 | C/1 | C/1 | A/0 | A/0 | A/0 |
| 71 | 71.3 | Image-based | E | E/1 | E/1 | E/1 | E/1 | E/1 | E/1 |
| 71 | 71.4 | Image-based | D | D/1 | D/1 | A/0 | D/1 | D/1 | D/1 |
| 71 | 71.5 | Image-based | C | C/1 | C/1 | C/1 | C/1 | D/0 | B/0 |
| 72 | 72.1 | Image-based | D | D/1 | D/1 | D/1 | D/1 | D/1 | A/0 |
| 72 | 72.2 | Image-based | B | B/1 | B/1 | B/1 | D/0 | B/1 | D/0 |
| 73 | 73.1 | Image-based | B | A/0 | B/1 | A/0 | B/1 | B/1 | B/1 |
| 73 | 73.2 | Nonimage-based | A | A/1 | A/1 | A/1 | A/1 | A/1 | A/1 |
| 74 | 74 | Image-based | A | A/1 | A/1 | B/0 | A/1 | C/0 | A/1 |
| 75 | 75.1 | Image-based | B | B/1 | B/1 | B/1 | B/1 | A/0 | B/1 |
| 75 | 75.2 | Image-based | D | B/0 | B/0 | B/0 | B/0 | B/0 | B/0 |
| 75 | 75.3 | Nonimage-based | D | D/1 | D/1 | D/1 | D/1 | B/0 | B/0 |
| 75 | 75.4 | Image-based | A | C/0 | A/1 | A/1 | A/1 | D/0 | B/0 |
| 76 | 76.1 | Image-based | D | D/1 | D/1 | D/1 | C/0 | D/1 | B/0 |
| 76 | 76.2 | Image-based | B | B/1 | B/1 | B/1 | B/1 | B/1 | B/1 |
| 76 | 76.3 | Nonimage-based | D | D/1 | D/1 | D/1 | D/1 | D/1 | B/0 |
| 77 | 77.1 | Image-based | D | D/1 | D/1 | D/1 | D/1 | D/1 | B/0 |
| 77 | 77.2 | Image-based | A | A/1 | A/1 | A/1 | A/1 | A/1 | B/0 |
| 78 | 78.1 | Image-based | B | B/1 | B/1 | B/1 | B/1 | A/0 | B/1 |
| 78 | 78.2 | Nonimage-based | E | C/0 | E/1 | E/1 | B/0 | E/1 | B/0 |
| 78 | 78.3 | Image-based | D | D/1 | D/1 | D/1 | A/0 | E/0 | B/0 |
| 78 | 78.4 | Nonimage-based | B | C/0 | C/0 | C/0 | D/0 | C/0 | B/1 |
| This de-identified table provides sequential case/question identifiers, image-based versus nonimage-based classification, specialist-reviewed reference answers, and final model-level responses based on the three-run majority rule. In model columns, values are shown as final answer/correctness flag, where 1 = correct and 0 = incorrect. | | | | | | | | | |
